# A latent growth curve model to estimate electronic screen use patterns amongst adolescents aged 10 to 17 years

**DOI:** 10.1186/s12889-018-5240-0

**Published:** 2018-03-07

**Authors:** Michael Rosenberg, Stephen Houghton, Simon C. Hunter, Corinne Zadow, Trevor Shilton, Lisa Wood, David Lawrence

**Affiliations:** 1 University of Western Australia, Faculty of Science, (M408) 35 Stirling Highway, Perth, WA Australia; 2 University of Western Australia, Faculty of Arts, Business, Law, and Education, 35 Stirling Highway, Perth, WA Australia; 3 University of Strathclyde, School of Psychological Sciences and Health, 40 George Street, Glasgow, UK; 4 University of Western Australia, Faculty of Medical Sciences, 35 Stirling Highway, Perth, WA Australia; 5 National Heart Foundation of Australia, 334 Rokeby Road, Subiaco, Perth, WA Australia

**Keywords:** Adolescents, Electronic screen use, Guidelines

## Abstract

**Background:**

High quality, longitudinal data describing young people’s screen use across a number of distinct forms of screen activity is missing from the literature. This study tracked multiple screen use activities (passive screen use, gaming, social networking, web searching) amongst 10- to 17-year-old adolescents across 24 months.

**Methods:**

This study tracked the screen use of 1948 Australian students in Grade 5 (*n* = 636), Grade 7 (*n* = 672), and Grade 9 (*n* = 640) for 24 months. At approximately six-month intervals, students reported their total screen time as well as time spent on social networking, passive screen use, gaming, and web use. Patterns of screen use were determined using latent growth curve modelling.

**Results:**

In the Grades 7 and 9 cohorts, girls generally reported more screen use than boys (by approximately one hour a day), though all cohorts of boys reported more gaming. The different forms of screen use were remarkably stable, though specific cohorts showed change for certain forms of screen activity.

**Conclusion:**

These results highlight the diverse nature of adolescent screen use and emphasise the need to consider both grade and sex in future research and policy.

## Background

Electronic screens are now a ubiquitous part of the adolescent landscape, occupying a large amount of their daily time, [1–10] and becoming an important aspect of their lives [11]. Today, virtually all 8 to 17-year-olds in developed countries access the internet at home, school or at their friends’ homes and increasingly through mobile devices outside of these settings [4, 9, 11]. Data suggest US children older than eight years of age spend on average 6.4 h per day on screen-based activities [see 9]. In Australia, electronic media use amongst 11–13-year-olds averages 4.8 h on a week day and 5.8 h on a weekend day [5], with an average of 3.8 h a day spent on sedentary screen time [12]. Access to multiple portable and constantly connected devices continues to proliferate, and today’s mobile phones can be simultaneously an internet, gaming and social media device. The consumption of multiple screen based activities contributes towards a high prevalence of screen use amongst children and adolescents [4, 5, 9, 13, 14].

Some studies suggest screen use has been associated with a range of adverse physical [15–19] and mental health outcomes [20–23]. However, the more recent research literature has debated the extent to which screen use is associated with these health related issues. For example, two separate meta-analyses have reported only small effects of video game use on behavioural and mental health outcomes [24] and no effects of TV and video game use with body fatness [25]. However, the relationships between screen use and positive and negative behavioural related outcomes may be more complex. That is, screen use up to one hour per day can be beneficial, but more extensive use may be detrimental [26]. In line with this, four linked studies have suggested that young people need to reach critical cut-off points before negative effects are observed [27]. The American Academy of Pediatrics (AAP) recently recognised both the positive and negative effects of screen use on young people by recommending that parents develop personalised media use plans with their children so as to achieve a balance in lifestyle choices [28, 29].

A related issue concerns the extent to which national guidelines for youth ignore the diversity of screen use [30]. Typically, electronic media for entertainment has focused on use for recreation, combining TV viewing, gaming, and computer use as non-interactive activities [9]. This neglects the social active-engagement that now co-exists with a wide range of screen-based activities [9]. Given the plethora of devices, content, and connection, and normalisation of screen use, understanding young people’s use of screens over time is crucial. High quality, contemporary, longitudinal data which describes young people’s screen use across a number of distinct forms of screen activity is very limited in the literature. This is not to say that no longitudinal evidence exists. Higher amounts of initial screen time were found to be significantly associated with higher initial symptoms of depression in one study [20], whereas no predictive main effect of screen time on depressive symptoms (one year later) was found in another [31]. While such information is important when trying to understand screen use among youth, more research is needed to obtain a comprehensive picture about trends in screen use and separate screen use activities over time. Therefore, the purpose of this present study was to track patterns of multiple screen use activities (passive screen use, gaming, social networking, web searching) amongst 10- to 17-year-old adolescents using a latent growth curve model.

## Method

### Research design

This research utilised an accelerated longitudinal cohort sequential design (ALCSRD) to assess and represent change in screen use spanning approximately 7 years (10- to 17-years of age). The ALCSRD links adjacent segments of limited longitudinal data from different age cohorts to estimate a common long-term developmental trend, or growth curve. Thus, researchers can approximate a long-term longitudinal study by conducting several simultaneous short-term longitudinal studies of different age cohorts [32]. The validity of the ALCSRD as a valid and effective means of constructing the full longitudinal growth curve has been demonstrated for youth [33–37] and health-related behaviours, including physical activity [32, 38].

### Participants

As shown in Table 1, three cohorts of participants were randomly recruited at Time 1 (T1) from Grade 5 (10/11 years of age: 346 males, 307 females), Grade 7 (12/13 years of age: 370 males, 324 females), and Grade 9 (14/15 years of age: 356 males, 312 females). These cohorts were retested on a further five occasions over three academic/calendar school years (2013–2015). To be included in the final analysis participants were required to have completed the initial survey (T1). Consequently, a total of 1948 children and adolescents (1030 males and 918 females) were retained for analysis.

**Table 1:** Mean (SD) Hours of Screen Use at Each Time Point, by Cohort

Screen Use Measure	Time 1^1^	Time 2^2^	Time 3^3^	Time 4^4^	Time 5^5^	Time 6^6^
Overall						
Grade 5	3.05 (2.32)	2.65 (2.16)	2.60 (2.13)	2.60 (2.12)	2.89 (2.22)	3.02 (2.20)
Grade 7	3.92 (2.44)	3.78 (2.61)	3.67 (2.28)	3.89 (2.47)	3.92 (2.38)	4.04 (2.43)
Grade 9	4.95 (2.70)	4.53 (2.47)	4.57 (2.44)	4.66 (2.63)	4.63 (2.60)	4.48 (2.47)
Social Networking						
Grade 5	0.88 (1.77)	0.86 (1.84)	0.76 (1.40)	0.93 (1.75)	1.18 (2.00)	1.30 (1.99)
Grade 7	1.70 (2.45)	1.71 (2.51)	1.58 (2.17)	1.72 (2.41)	1.82 (2.33)	1.93 (2.32)
Grade 9	2.52 (2.99)	2.17 (2.58)	2.20 (2.56)	2.28 (2.71)	2.48 (2.69)	2.19 (2.50)
Gaming						
Grade 5	2.09 (2.28)	1.86 (2.26)	1.64 (2.06)	1.60 (1.94)	1.41 (2.02)	1.38 (1.97)
Grade 7	1.88 (2.16)	1.71 (2.43)	1.27 (1.85)	1.43 (2.10)	1.29 (2.24)	1.23 (2.10)
Grade 9	1.62 (2.33)	1.57 (2.37)	1.40 (2.22)	1.42 (2.43)	1.31 (2.57)	1.17 (2.29)
Web						
Grade 5	1.46 (1.93)	1.35 (1.81)	1.26 (1.65)	1.25 (1.64)	1.46 (1.64)	1.34 (1.47)
Grade 7	1.73 (1.99)	1.90 (2.15)	1.64 (1.64)	1.84 (2.10)	1.78 (1.94)	1.79 (1.95)
Grade 9	2.09 (2.17)	2.04 (2.14)	1.87 (2.25)	2.17 (2.20)	2.42 (2.50)	2.15 (2.42)
Passive						
Grade 5	2.45 (2.28)	2.18 (2.17)	2.09 (1.96)	2.14 (2.11)	2.06 (1.99)	2.04 (1.89)
Grade 7	2.73 (2.41)	2.80 (2.52)	2.35 (2.01)	2.47 (2.33)	2.41 (2.27)	2.48 (2.28)
Grade 9	3.02 (2.59)	2.84 (2.32)	2.69 (2.43)	2.73 (2.53)	2.59 (2.57)	2.56 (2.65)

^1^*N* = 590 to 652. ^2^*N* = 481 to 589. ^3^*N* = 461 to 539. ^4^*N* = 476 to 541. ^5^*N* = 373 to 483. ^6^*N* = 378 to 462

Participants were recruited from 25 randomly selected schools comprising state government primary schools (K-6) and high schools (7–12) (4 in rural locations), and non-government schools (K-12). All schools were located across a range of socio-economic status (SES) areas as indexed by their Socio-Economic Index for Areas (SEIFA), Australia, 2011 [39] and spread across metropolitan and regional areas. Initially schools were identified from different rural and metro geographical locations and SES areas across Western Australia in an attempt to ensure that any sample generated would be representative of the wider WA school and student population. A number of schools from each of these locations and SES areas were invited to participate. Of the 25 schools contacted, all agreed to involved.

### Instrumentation

The Screen Based Media Use Scale (SBMUS), detailed in Houghton et al. (2015) [9] is an online instrument designed to collect data on “total time spent on screens” and types of screen activities. What ‘screens’ refer to and what they are typically used for is first clarified in the SBMUS (images are shown of iPod Touch, iPad, Mobile Phone, TV, Laptop, Computer, Xbox). Examples of screen activities are then provided. Participants are asked to estimate their overall use of screens on a typical week day from waking up until the time they go to bed (including use both during and outside of school) using an interactive slide bar that measures screen use in hours and minutes. This is then repeated for a typical weekend day. An overall screen use score for average weekly use was created by using a weighted average.

Four separate sections of questions on gaming, social networking and instant messenger, T*V*/Videos/Music (passive screen use), and web use are then presented. Each section requires participants to use an interactive slide bar to estimate their SBMU in hours and minutes [as previously for total time spent on screens].

Previous research [9] has demonstrated the SBMUS has satisfactory test-retest reliability across a 6-month period: Overall reliability (*r* = .50, *N* = 174) and this did not differ by sex (r(boys) = .51, *n* = 91; r(girls) = .53, *n* = 82). To ensure comprehensive coverage of screen use across the six administrations, surveys were administered at different times: T1 August/September 2013; T2 November/December 2013; T3 March/April 2014; T4 August/September 2014; T5 March/April 2015; and T6 August/September 2015.

### Procedure

Permission to conduct this research was obtained from the Human Research Ethics Committees of the University of Western Australia and the State Department of Education. Data collection was carried out following the principles outlined in the Declaration of Helsinki. We obtained written consent from principals, parents and students and reminded students (assent) on the day of participation via their teacher that if they wished they could withdraw from the study without prejudice. The SBMUS was administered to participants via an online survey during regular school hours. If a student was absent on any of the data collection days, an alternative time was arranged by the school to ensure survey completion.

### Data analysis

MPlus version 7.31 [40] was used to estimate all latent growth curve models (LGCMs). This technique estimates latent variables corresponding to growth parameters (slope and intercept) [32, 34, 41]. Full information maximum likelihood (FIML) was used in the analyses to address missing data and the MLR estimator was used in order to address non-normality.

**Fig. 1:**
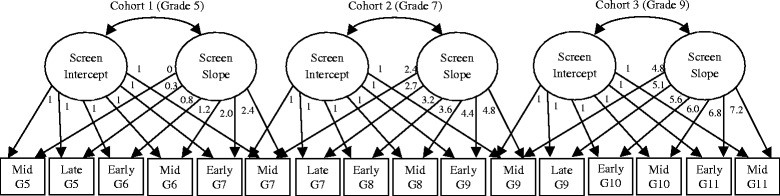
Representation of the cohort-sequential LGCM

The analyses described here were repeated five times, i.e., for total screen time, gaming, social networking, web use, and passive screen use. First, a cohort-sequential LGCM [34] was conducted where the three cohorts were used to estimate a common intercept and slope, modelling screen use from mid-Grade 5 to mid-Grade 11. This was an unconditional growth model with random intercept and random slope: Each cohort accounted for a different section of the overall slope, and overlap in sampling (at Grades 7 and 9) allowing the adequacy of a model describing a common developmental trend to be assessed (see Fig. 1). The loadings of observed variables on the slope variable were constrained to reflect the timing (in months) of each data collection point, had a true longitudinal design been used. These loadings are divided by 10 in order to avoid convergence problems associated with large time scores, hence they varied from 0 to 7.2 instead of 0 to 72 [40]. The overlapping data collection points (mid-Grade 7, mid-Grade 9) were loaded as the same weight in both cohorts sharing that time point. In this way, a common LGCM was estimated from mid-Grade 5 to mid-Grade 11.

**Fig. 2:**
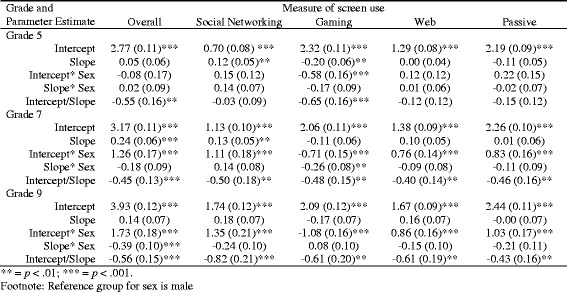
Representation of the multi-group LGCM

A cohort-specific LGCM approach (see Fig. 2) then assessed whether individual LGCMs for each cohort better accounted for the data than the common slope estimated in the cohort-sequential LGCM. This allowed for the possibility of differences in slopes and intercepts across the three cohorts (Grade 5, Grade 7, and Grade 9). Loadings of observed variables on the intercept latent variable were constrained to 1. Loadings of the observed variables on the slope latent variable were constrained to 0, 0.3, 0.8, 1.2, 2.0, and 2.4 to reflect the timing (in months) of each data collection point (again, divided by 10). This was again a random intercepts, random slopes model. Here, the means and variances of the slope and intercept were allowed to vary across the three cohorts, as was the slope-intercept covariance. The cohort-sequential LGCMs and the cohort-specific LGCMs were compared using the chi-square difference test (Δχ^2^): Significant differences indicate that the model with additional constraints (the cohort-sequential LGCM) should be rejected.

For all five measures of screen use, the cohort-specific model was a better fit to the data than the cohort-sequential model and there was significant variation in both intercept and slope [41]. We further evaluated a final model where the effects of sex upon slope and intercept were assessed. This model was a conditional cohort-specific LGCM, with both intercept and slope predicted by sex.

## Results

### Loss to follow-up

There were 1948 participants at Time 1. Across subsequent data collection points, this reduced to 1705 at T2 (12.5% attrition), 1597 at T3 (18.0% attrition), 1578 at T4 (19.0% attrition), 1337 at T5 (31.4% attrition), and 1303 at T6 (33.1% attrition). To understand if attrition was associated with sex, Grade, or rural/urban status, we conducted a logistic regression predicting participant presence or absence at T6. Nagelkerke R^2^ for the regression model was .010. Participants lost to follow-up were more likely to be girls than boys (AOR = 1.37, 95% CI [1.14, 1.66], *p* = .001), no other predictors were detected.

### Descriptive statistics

Reported time spent on each form of screen use differed quite substantially over time (see Table 1). Total screen time was estimated at approximately 3 h per day by the Grade 5 cohort, 4 h per day by the Grade 7 cohort, and 4.5 to 5 h a day in the Grade 9 cohort. Social networking generally appeared to increase from T1 to T6, gaming and passive screen use decreased, and web use was relatively stable.

### Results of latent growth curve analyses

The fit of the cohort-sequential latent growth curve models (LGCM) were relatively poor across all five measures, with weaknesses on certain indices of fit (see Table 2). In addition, the model failed to converge for social networking. The fit indices for the cohort-specific LGCMs were good across all five measures and the variances for all intercept and slope estimates were significant (all *p* < .001). The Δχ^2^ tests supported the superiority of the second models over the first. The conditional cohort-specific LGCMs which included sex also demonstrated good fit, and these were selected as the final models. Parameter estimates of the conditional cohort-specific LGCMs are reported in Table 3. Since five models were estimated, we applied a Bonferroni correction, hence only effects significant at the 99% level (*p* < .01) were interpreted.

**Table 2:** Fit Indices of all Models, and Δχ2 Test Results of Relevant Model Comparisons

Variable	Model	χ^2^ (df)	CFI	RMSEA (90% CI)	SRMR	Δχ^2^ (df) to previous
Overall	(1) Full Accelerated LGCM	286.53 (60), *p* < .001	.858	.076 (.068, .085)	.113	N/A
	(2) Multi-Group LGCM	98.20 (48), *p* < .001	.969	.040 (.029, .051)	.048	188.33 (12), *p* < .01
	(3) Sex Added to (2)	121.55 (60), *p* < .001	.968	.040 (.030, .050)	.044	N/A
Social Networking	(1) Full Accelerated LGCM	Does not converge*				
	(2) Multi-Group LGCM	79.30 (48), *p* = .003	.968	.032 (.019, .044)	.045	N/A
	(3) Sex Added to (2)	105.36 (60), *p* < .001	.964	.034 (.023, .045)	.042	N/A
Gaming	(1) Full Accelerated LGCM	160.40 (60), *p* < .001	.887	.051 (.041, .060)	.098	N/A
	(2) Multi-Group LGCM	81.61 (48), *p* < .001	.962	.033 (.020, .045)	.053	78.79 (12), *p* < .01
	(3) Sex Added to (2)	98.44 (60), *p* = .001	.967	.031 (.020, .042)	.049	N/A
Web use	(1) Full Accelerated LGCM	151.08 (60), *p* < .001	.883	.048 (.039, .058)	.106	N/A
	(2) Multi-Group LGCM	74.51 (48), *p* = .008	.966	.029 (.015, .042)	.052	76.57 (12), *p* < .01
	(3) Sex Added to (2)	94.29 (60), *p* = .003	.965	.030 (.017, .041)	.048	N/A
Passive	(1) Full Accelerated LGCM	185.52 (60), *p* < .001	.872	.057 (.048, .066)	.092	N/A
	(2) Multi-Group LGCM	75.48 (48), *p* = .007	.972	.030 (.016, .042)	.045	110.04 (12), *p* < .01
	(3) Sex Added to (2)	93.23 (60), *p* = .004	.972	.029 (.017, .040)	.041	N/A

*Covariance matrix (PSI) in one or more groups is not positive definite

Good model fit = CFI values above .95; RMSEA scores of .06 or less; SRMR <.05

**Table 3:** Unstandardized Parameter Estimates (with Standard Errors) for Conditional Multi-Group LGCMs

Grade and Parameter Estimate	Measure of screen use				
	Overall	Social Networking	Gaming	Web	Passive
Grade 5					
Intercept	2.77 (0.11)***	0.70 (0.08)***	2.32 (0.11)***	1.29 (0.08)***	2.19 (0.09)***
Slope	0.05 (0.06)	0.12 (0.05)**	−0.20 (0.06)**	0.00 (0.04)	− 0.11 (0.05)
Intercept* Sex	−0.08 (0.17)	0.15 (0.12)	−0.58 (0.16)***	0.12 (0.12)	0.22 (0.15)
Slope* Sex	0.02 (0.09)	0.14 (0.07)	− 0.17 (0.09)	0.01 (0.06)	− 0.02 (0.07)
Intercept/Slope	−0.55 (0.16)**	−0.03 (0.09)	− 0.65 (0.16)***	−0.12 (0.12)	− 0.15 (0.12)
Grade 7					
Intercept	3.17 (0.11)***	1.13 (0.10)***	2.06 (0.11)***	1.38 (0.09)***	2.26 (0.10)***
Slope	0.24 (0.06)***	0.13 (0.05)**	−0.11 (0.06)	0.10 (0.05)	0.01 (0.06)
Intercept* Sex	1.26 (0.17)***	1.11 (0.18)***	−0.71 (0.15)***	0.76 (0.14)***	0.83 (0.16)***
Slope* Sex	−0.18 (0.09)	0.14 (0.08)	−0.26 (0.08)**	−0.09 (0.08)	− 0.11 (0.09)
Intercept/Slope	−0.45 (0.13)***	− 0.50 (0.18)**	−0.48 (0.15)**	− 0.40 (0.14)**	−0.46 (0.16)**
Grade 9					
Intercept	3.93 (0.12)***	1.74 (0.12)***	2.09 (0.12)***	1.67 (0.09)***	2.44 (0.11)***
Slope	0.14 (0.07)	0.18 (0.07)	−0.17 (0.07)	0.16 (0.07)	−0.00 (0.07)
Intercept* Sex	1.73 (0.18)***	1.35 (0.21)***	−1.08 (0.16)***	0.86 (0.16)***	1.03 (0.17)***
Slope* Sex	−0.39 (0.10)***	−0.24 (0.10)	0.08 (0.10)	−0.15 (0.10)	− 0.21 (0.11)
Intercept/Slope	−0.56 (0.15)***	− 0.82 (0.21)***	−0.61 (0.20)**	− 0.61 (0.19)**	−0.43 (0.16)**

** = *p* < .01; *** = *p* < .001

Footnote: Reference group for sex is male

### Overall screen time

**Fig. 3:**
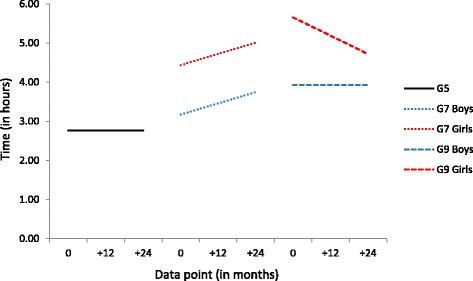
Conditional, multi-group Latent Growth Curve Model results for overall screen time

Sex had no effect on either the slope or intercept amongst the Grade 5 cohort, with overall screen time estimated at 2 h 46 min at the start of the study, remaining flat across the subsequent 24 months (see Fig. 3). Both boys and girls in the Grade 7 cohort reported increasing overall screen time across the 24 months, although at all time points girls reported an average of 1 h 16 min more than boys (girls_T1_ = 4 h 26 min, boys_T1_ = 3 h 10 min). Among the Grade 9 cohort, girls reported the highest overall screen use of any sub-group (5 h 40 min) which then decreased across the 24 months, whereas boys started at a lower level than girls (3 h 56 min) and remained at that level across the data collection period.

### Social networking

**Fig. 4:**
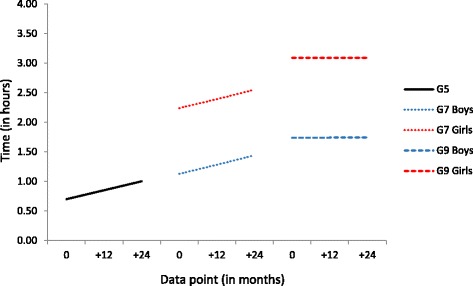
Conditional, multi-group Latent Growth Curve Model results for time spent social networking

Sex had no effect on either the slope or intercept amongst the Grade 5 cohort, and while the time spent on social networking started at 42 min per day this increased by 7 min per month across T1 to T6 (see Fig. 4). Among the Grade 7 cohort, girls reported more frequent daily social networking at T1 (2 h 14 min) than boys (1 h 8 min) and both boys’ and girls’ social networking subsequently increased by 8 min per month. For the Grade 9 cohort, girls (3 h 5 min) again reported more frequent social networking than boys (1 h 44 min) at T1, though there was no subsequent increase in levels of social networking reported.

### Gaming

**Fig. 5:**
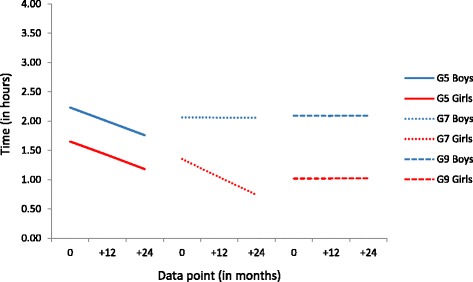
Conditional, multi-group Latent Growth Curve Model results for time spent gaming

In the Grade 5 cohort, boys reported gaming more than girls at T1 (2 h 14 min and 1 h 39 min daily, respectively), and this decreased at an equal rate for both sexes across T1 to T6 (see Fig. 5). Boys in the Grade 7 cohort also reported more time spent gaming than girls at T1 (2 h 4 min and 1 h 21 min, respectively), and girls (but not boys) decreased their subsequent time spend gaming. In the Grade 9 cohort, boys again reported more frequent gaming than girls (2 h 5 min and 1 h 1 min, respectively) and neither sex reported subsequent changes in time spent gaming.

### Web use

**Fig. 6:**
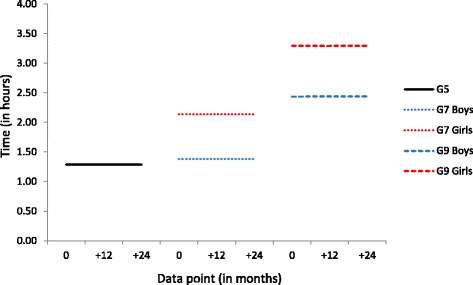
Conditional, multi-group Latent Growth Curve Model results for time spent on web use

Levels of web use remained stable from T1 to T6 within each cohort. In the Grade 5 cohort, both boys and girls reported using the web for 1 h 17 min per day (see Fig. 6). Girls reported more frequent web use than boys for both the Grade 7 cohort (2 h 8 min and 1 h 23 min, respectively) and the Grade 9 cohort (3 h 17 min and 2 h 26 min, respectively).

### Passive screen use

**Fig. 7:**
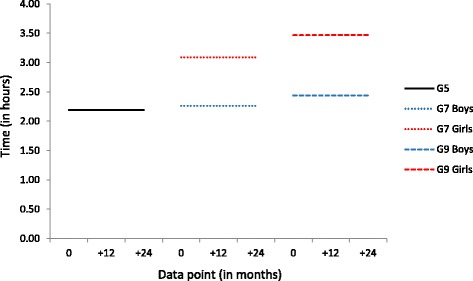
Conditional, multi-group Latent Growth Curve Model results for time spent on passive screen use

As for web use, levels of passive screen use remained stable from T1 to T6 within each cohort. There was no sex difference in frequency of passive screen use in the Grade 5 cohort (2 h 11 min) (see Fig. 7). In the Grade 7 cohort, girls reported more frequent passive screen use than boys (3 h 5 min and 2 h 16 min daily, respectively). In the Grade 9 cohort, girls again reported more frequent passive screen use than boys (3 h 28 min and 2 h 26 min, respectively).

## Discussion

This study has identified important nuances in patterns of screen use at different ages, emphasizing the heterogeneous nature of adolescent screen use. Screen access is ubiquitous amongst today’s adolescents [28, 42–44] and our results reinforce the high level of screen time 10–17 year olds engage on a daily basis [9, 21]. In the present study, we observed several different forms of screen use to be remarkably stable, though specific cohorts showed change for certain forms of screen activity. This stable pattern has not previously been reported in the literature, and may reflect important differences in the ways in which the rapidly changing electronic landscape is negotiated by each new set of young people [45]. In Grades 7 and 9 girls engage more than boys in all activities except gaming. Although Grade 9 boys’ estimates of total screen time remains stable for 24 months this is not the case for Grade 9 girls who have higher screen use which decreases over time. These results suggest that Grades 7 to 11 are periods when screen use is most fluid, implying screen use may be more amenable to change at these times, and efforts to change or moderate screen behaviours may be best targeted before these periods of significant increase in screen use.

The accelerated longitudinal cohort sequential research design utilised here was employed in the expectation that a unitary rate of change for each form of screen use could be estimated across the grade levels sampled (Grades 5 to 11). However, our results indicate that there were important cohort effects meaning that, for example, when the Grade 5 baseline cohort reached Year 7 they displayed different patterns of screen use to the Grade 7 baseline cohort. These cohort effects are likely to reflect the rapidly changing and evolving technologies available to young people: smartphone ownership among adolescents increased from 23% in 2011, to 37% in 2013 [45], and then 73% in 2015 [46]. Similarly, tablet computing has increasingly become a common activity in school classrooms [47]. Such developments can fundamentally change the ways in which young people use screens. For example, adolescents with smartphones (as compared to adolescents with cell phones) text more, and rely on social media and phone calls less, when contacting close friends [48]. The pace of change in technology therefore makes it very likely that each successive cohort of young people will experience and interact with the digital landscape in new and novel ways. In this rapidly evolving environment, clearly documenting young people’s changing screen use is important.

The pattern of screen use observed amongst participants in this study suggests that time spent in each main screen activity is relatively stable, across 24 months, within each cohort. The exceptions are declines in gaming amongst all Year 5 s and Year 7 girls, and increases in social networking among all Grade 5 s and Grade 7 s. The overall screen use trends resonate with previous findings that gaming declines with age, particularly for girls [49]. Our results support findings that social networking is more heavily used by girls than by boys [50] though suggest that this emerges only in Grade 7 (around 12 or 13 years old). Certainly, there were high levels of social networking reported, especially by girls, suggesting it is a highly normative and compelling activity. This is likely to make any effort to reduce or moderate screen use relating to this activity particularly challenging. The average time spent on each type of screen use summed across the four categories used in this study was substantially higher than the average total time spent using screens in each cohort, and for each time period. This reflects a high degree of multi-tasking and multiple screen use. Young people frequently multitask, using several screens at the same time (e.g. playing a game of using social media on a smartphone or tablet at the same time as watching TV or videos on the web.

Screen use amongst adolescents is now an entrenched and dominant sedentary behaviour [9, 28, 51] with data from this study suggesting that older adolescents spend considerable portions of their waking time engaged in screen activity. In fact, screen use may account for approximately 35% of girls’, and 25% of boys’, waking time (based on an average of 8 h sleep). However, contemporary research argues for a focus on a range of forms of screen use [9] rather than focussing on a single form (e.g., gaming) or on a single measurement of overall time spent on screens. The differences in screen time we have documented here support such an approach in future.

This study has a number of advantages when compared to the existing literature, including explicit assessment of the development of screen use across multiple cohorts across a two-year period. This design is unique in this literature and has highlighted important cohort effects which have not been documented before. However, limitations of the study should be considered when interpreting the results. The study relied upon self-report of screen use and activities, and it was not feasible to conduct objective validation of these. While such validation is desirable, it is extremely challenging for a number of reasons including the use of screens at school, the number of screens used by young people, and the use of screens which are not the participants’ own (e.g., friends’ or other family members’ smartphones, tablets, TVs etc.).

There was some disparity between the participants estimations of the total time they spent using screens compared to the time they spent on each of the four screen activities. Young people often undertake more than one screen activity simultaneously (e.g. using social media on their phone at the same time as watching TV) and therefore by simply adding each screen activity estimate to generate a total screen time is not an accurate method of estimation.

## Conclusions

In conclusion, these results highlight the heterogeneity of adolescent screen use, and in particular the merits of delineating the type of screen activity, rather than just focusing on overall screen time [9], as presented in the AAP updated recommendations [29]. Future guidelines may consider significant changes in screen media consumption patterns across adolescence. If effective public health communications are to be developed, future refinement of screen use guidelines and recommendations need to directly consider how they can be flexible enough to respond to both cohort-level and shifts in young people’s screen based behaviours and experiences.

## Abbreviations

AAP: American Academy of Pediatrics
ALCSRD: Accelerated longitudinal cohort sequential design
FIML: Full information maximum likelihood
K-12: Kindergarten to Year 12
K-6: Kindergarten to Year 6
LGCM: Latent growth curve models
SBMUS: Screen based Media Use Scale
SEIFA: Socio-Economic Index for Areas
SES: Socio-economic status
